# Sinapic Acid Attenuates Cardiovascular Disorders in Rats by Modulating Reactive Oxygen Species and Angiotensin Receptor Expression

**DOI:** 10.1155/2020/1436858

**Published:** 2020-07-11

**Authors:** Maha A. Aldubayan, Amira S. Ahmed, Ashraf M. Emara, Ahmed A. Ahmed, Rehab M. Elgharabawy

**Affiliations:** ^1^Department of Pharmacology & Toxicology, College of Pharmacy, Qassim University, Qassim, Saudi Arabia; ^2^Hormones Department, Medical Research Division, National Research Centre, Giza, Egypt; ^3^Department of Forensic Medicine & Clinical Toxicology, Faculty of Medicine, Tanta University, Tanta, Egypt; ^4^Medical Research Center, Faculty of Medicine, Qassim University, Qassim, Saudi Arabia; ^5^Department of Pharmacology & Toxicology, Faculty of Pharmacy, Tanta University, Tanta, Egypt

## Abstract

The main avoidable risk factor for cardiovascular conditions is high blood pressure (hypertension). At global level, hypertension is believed to be responsible for a 54% stroke-related mortality rate and a 47% mortality rate associated with coronary heart disease. It is postulated that sinapic acid (SA) could help in hypertension management because it displays robust antioxidant, antihyperglycemic, and peroxynitrite scavenging effects. To explore this hypothesis, this work examined the effect of SA on oxidative stress and cardiovascular disease in rats with hypertension by comparison against captopril. For this purpose, 50 male rats were used and equally allocated to five groups, namely, normal control, positive control (L-NAME), L-NAME with concomitant captopril administration, L-NAME with concomitant SA administration, and L-NAME with concomitant administration of both SA and captopril. Results showed that, by contrast to control, L-NAME exhibited marked elevation in serum CK-MB, total cholesterol, triglycerides, VLDL-C, LDL-C, Ang II, AT2R, ET-1, and angiopoietin-2; on the other hand, L-NAME exhibited marked reduction in serum HDL-C, superoxide dismutase (SOD) activity, nitric oxide synthase 3 (NOS3), and glutathione (GSH). Furthermore, joint administration of SA and captopril ameliorated hypertension, enhanced cardiovascular function, hindered hyperlipidemia, and decreased oxidative stress and myocardial hypertrophy displayed by rats with hypertension. Based on such findings, better chemopreventive or therapeutic approaches can be devised to manage hypertension and cardiovascular conditions.

## 1. Introduction

The force of the blood against the walls of arteries during its circulation through the body is known as the blood pressure. When the blood is pumped continuously through the blood vessels with an inordinate amount of force, the blood pressure is classified as high (hypertension) [1]. Hypertension is a primary risk factor for a range of conditions, including stroke, coronary artery disease (CAD), myocardial infarction, kidney disease, heart failure, and peripheral vascular disease, which is why it is a major issue for public health [2]. Indeed, in both developed and developing nations, hypertension is associated with a high mortality and morbidity rate because it is widely prevalent and can heighten the likelihood of cardiovascular disease (CVD) and atherosclerosis [3, 4]. According to statistics from the World Health Organization (WHO), by 2030, the mortality rate associated with CVD is anticipated to be around 23.6 million individuals [5]. At the same time, blood pressure and CVD risk factors are influenced by lifestyle and diet [6].

At global level, over 600 million individuals suffer from hypertension and 13% of them die from this condition. The percentage of the global adult population with hypertension is expected to reach 29% by 2025 [7]. As a result, high mortality rates are associated with CVDs such as heart toxicity, hypertension, and heart failure, as well as stroke [8, 9].

Angiotensin-converting enzyme (ACE) inhibitors are predominately administered to the patients with hypertension because of their decreasing effect on vascular resistance and blood pressure without considering cardiac condition. ACE inhibitors have an improving cardiac effect in patients with myocardial ischemia and hypertrophy without relation with their regulating effect on blood pressure [10].

ACE inhibitors affect the local renin-angiotensin system in many tissues. ACE inhibitors do their effects through two main mechanisms. They suppress the production of angiotensin II that elevates inotropy and chronotropy and subsequently interact with sympathetic activation. After long-term duration, angiotensin II may play like a growth factor within the heart tissue. Suppression of angiotensin II production is associated with accumulation of angiotensin fragments that cause vasodilatation of the coronary arteries. The other main mechanism of ACE inhibitors is decreasing the metabolism of bradykinin. Bradykinin increases blood flow in the coronary arteries and inotropy and corrects endothelial functions through decreasing the metabolism of energy-rich phosphates in patients with myocardial ischemia and hypertrophy. Bradykinin stimulates the release of potent vasoactive modulators such as nitric oxide (NO), prostaglandins, and endothelium-derived hyperpolarizing factor [11].

There is evidence that considerable action against oxidants is exhibited by a broad range of plant extracts and related products. This property could be exploited to manage CVDs and other conditions with a high mortality rate [12–14]. The phenolic compound contained in fruit and vegetables is the primary reason why eating such products can reduce the risk of CVDs [15, 16].

Spices, citrus and berry fruits, vegetables, cereals, oilseed crops, and other edible plants contain the phytochemical known as sinapic acid (SA) [17]. Derived from cinnamic acid, SA is classified as a phenolic acid characterised by 3,5-dimethoxyl and 4-hydroxyl substitutions in the cinnamic acid phenyl group. It displays various pharmacological and biological effects, including neuroprotective effect [18] and effect against microbes [19], inflammation [20], oxidants [21, 22], bacteria [17], and hyperglycemia [23], while also having antifungal potential [24]. These properties explain the growing attention paid to SA.

In the treatment of hypertension, combination therapy has more advantages than monotherapy, including complementary effects of antihypertensive mechanisms, superposition of efficacy, and ultimate reduction of adverse reactions.

As the latest discoveries suggest, hypertension, lipid metabolism changes, and other cardiovascular complications occur primarily as a result of oxidative stress. When reactive oxygen species (ROS) are generated in excess, oxidative stress can promote the development of different disorders, including cancer, neurodegenerative conditions, ageing, and CVDs [16, 25, 26]. Given the above considerations, this work employed L-NAME-induced rats with hypertension to determine how SA affected cardiac functions and structure as well as enzymatic antioxidants and lipid profile levels.

## 2. Materials and Methods

### 2.1. Chemicals

Sigma-Aldrich (St. Louis, Missouri, USA) supplied the chemicals needed for this study, namely, N*ω*-nitro-L-arginine methyl ester hydrochloride (L-NAME), SA, and captopril.

### 2.2. Experimental Animals

For research purposes, 50 albino Wistar male rats with a weight of 180-220 g were used. They were kept in cages made of wire mesh under conditions of 23°C ± 2°C temperature, 80% ± 5% relative humidity, and 12-hour alternating light and dark cycles. No restrictions were imposed on standard diet and water. The animal research protocols of the Ethical Committee of Faculty of Pharmacy, Qassim University, Saudi Arabia, were followed in conducting the empirical work.

### 2.3. Treatment Administration

A 40 mg/kg body weight (b.w.) of L-NAME and 40 mg/kg/day of captopril were, respectively, administered to the rats in the drinking water over a period of four weeks [27, 28]. An intragastric tube was employed for daily oral administration of SA dissolved in corn oil over a period of four weeks [29]. The rats were arbitrarily and equally distributed into five experimental groups as follows: the first group was the control group that was administered corn oil; the second group was administered 40 mg/kg b.w. of L-NAME; the third group was administered L-NAME alongside 40 mg//kg b.w. of SA; the fourth group was administered L-NAME alongside 40 mg/kg/day of captopril; and the fifth group was administered L-NAME alongside both 40 mg/kg b.w. SA and 40 mg/kg/day captopril.

### 2.4. Systolic Blood Pressure Measurement

The onset and development of hypertension were assessed with the tail-cuff method with a Narco Biosystems Electrosphygmomanometer after the rats were warmed at 35°C for 5 minutes, while under slight restraint. Blood pressure was measured under conscious conditions one day before the start of the experiment and at the end of the experiment.

### 2.5. Collection of Blood Samples

Once the empirical work was finished, the rats were administered sodium pentobarbital intraperitoneally to euthanise them. The blood was then sampled from the inferior vena cava of every rat and allowed to coagulate in nonheparinised glass tubes for half an hour at ambient temperature. Afterwards, the samples were subjected to 10-minute centrifugation at 5000 rpm. Separation of serum samples was also undertaken, and these were kept in aliquots at a temperature of -80°C to await future use. The samples were defrosted at ambient temperature before assay.

#### 2.5.1. Cardiac Biomarker Measurement

The Cloud-Clone Corp. Rat Troponin I ELISA kit (Cloud-Clone Crop., Houston, USA) was employed to conduct a quantitative sandwich immunoassay for determining the level of troponin I in the serum in keeping with the approach suggested by Penttilä et al. [30]. Meanwhile, the level of creatine kinase-MB enzyme in the serum (CK-MB) was determined with the assay kit supplied by Bioassay Systems (Hayward, CA, USA) and based on the method by Bishop et al. [31].

#### 2.5.2. Vascular Biomarker Measurement

The levels of angiotensin II (Ang II) and Ang II type 2 receptor (AT2R/AT2) in serum were, respectively, measured based on the approach by Volland et al. [32] and the manufacturer's guidelines via quantitative sandwich ELISA kits (Uscn Life Science Inc., Wuhan). Similarly, the levels of endothelin-1 (ET-1) and angiopoietin-2 in the serum were, respectively, measured based on the approach by Suzuki et al. [33] and the manufacturer's guidelines via Cloud-Clone Corp. ELISA kits.

#### 2.5.3. Oxidative Stress Biomarker Measurement

The method recommended by Tousson et al. [34] was adopted to measure the serum levels of enzymatic antioxidants such as superoxide dismutase (SOD) and nitric oxide synthase 3 (NOS3). The method by Emara et al. [35] was used to determine the level of reduced glutathione (GSH) in the serum.

#### 2.5.4. Lipid Profile Measurement

Reagent kits (Crescent Diagnostics Test, KSA) were used to apply enzymatic colorimetric techniques to measure the serum levels of total cholesterol in keeping with Salama et al. [1], triglycerides (TG) in keeping with Aldubayan et al. [36], and high-density lipoprotein cholesterol (HDL-C) in keeping with Bolkiny et al. [37]. As shown below, the equation proposed by Friedewald et al. [38] was applied to measure the levels of very low-density lipoprotein cholesterol (VLDL-C) and low-density lipoprotein cholesterol (LDL-C) in the serum:

(1) $$\begin{array}{c} LDL-C=\text{total cholesterol}–HDL-C–TG/5 \end{array}$$

### 2.6. Ang II Receptor Gene Expression

#### 2.6.1. Extraction of RNA

TissueRuptor (QIAGEN, Hilden, Germany) was employed for defrosting and homogenising cardiac tissue samples in RNA in QIAzol lysis solution (QIAGEN, Valencia, CA, USA) for 40 seconds at 33,000 rpm. The QuickGene automated system (QuickGene, USA) was implemented in keeping with the manufacturer's guidelines to extract the total RNA. RNase-free water was subsequently used for elution of RNA of high quality. Moreover, a Thermo Scientific NanoDrop spectrophotometer (Waltham, MA, USA) was employed to monitor the quality of RNA at 260 and 280 nm. Processing was conducted solely on the RNA samples with an absorbance ratio 260/280 nm of ~2.0>. The samples of total RNA were afterwards placed in storage at -70°C.

#### 2.6.2. Reverse Transcription (cDNA Synthesis)

The synthesis of single-stranded cDNA was conducted with 1.0 *μ*g total RNA and the High-Capacity cDNA Reverse Transcription Kit (NEB, USA). To synthesise first-strand cDNA, a first-strand cDNA synthesis kit was employed with a 24 T primer (0, 4 nmol/reaction) in a 25 *μ*l reaction mix, in line with the manufacturer's guidelines. Furthermore, before running in real-time PCR, tenfold dilution of the cDNA products was undertaken.

#### 2.6.3. Real-Time PCR (qPCR)

The qPCR core kit for SYBR Green (QIAGEN, USA) and StepOne Real-Time PCR System (Applied Biosystems, Foster City, CA) were employed to run the reaction of amplification with a 2.5× RT-PCR master mix. The 25 *μ*l mix used for every reaction consisted of 400 nmol of every primer and 1 *μ*l 1 : 10 diluted cDNA.  Table 1 provides the sequences of primers. Standard profile times were applied, namely, 15 minutes at 95°C in the first step, 15 seconds at 94°C in the second step, half minute at 60°C, and half minute at 72°C for 40 cycles with melting curve analysis. After normalisation to the equivalent glyceraldehyde 3-phosphate dehydrogenase (GAPDH) (Thermo Fisher, USA), the target mRNA was contrasted against untreated sample representing the control in the same plate. The 2^-*ΔΔ*CT^ technique was adopted to determine the values, with the discrepancy in threshold cycles for target and the housekeeping gene (GAPDH) being denoted by ΔCT and the discrepancy in ΔCT and the threshold cycles for the control being denoted by ΔΔCT.

### 2.7. Histopathology

The left heart ventricles from rats in different groups were surgically removed and cleaned of blood. A 10% neutral buffer formalin solution was afterwards used to fix the ventricles for two days, before they were subjected to processing for paraffin sectioning [39]. The method proposed by Tousson [34] was applied to stain sections with haematoxylin and eosin in preparation for histopathological analysis.

### 2.8. Statistical Analysis

The Statistical Package for the Social Sciences (SPSS; V. 21.0, Inc., USA) was used to conduct the statistical analysis of the data. The purpose of the statistical analysis was to determine the statistical significance of the linear trend noted across the L-NAME+SA, L-NAME+captopril, and L-NAME+SA+captopril groups. Statistical significance was indicated by a *P* value equal to or less than 0.05. The expression of the data took the form of mean ± SD. Unpaired *t*-test with Tukey's correction was carried out for ANOVA-based and post hoc pairwise comparisons of the various experimental groups. Furthermore, Pearson's correlation (*r*) was used to assess how the different parameters were correlated with one another, while the markers of CK-MB, troponin, ET-1, and ANG II were evaluated through the linear regression analysis. The chosen independent variables were the oxidative biomarkers NOS3 and GSH, the cardiac biomarkers CK-MB and troponin, and the vascular biomarkers ET-1 and ANG II.

## 3. Results

### 3.1. The Impact of SA and Captopril on Systolic Blood Pressure

The average baseline systolic blood pressure, measured 1 day before the start of the experiment, was 117 ± 0.9 mm Hg for all of the rats used in the experiment. The mean values of the systolic blood pressure of treated groups with L-NAME, L-NAME plus SA, L-NAME plus captopril, and L-NAME plus SA and captopril were 149 ± 4.8, 135 ± 3.7, 135 ± 3.7, and 114 ± 0.5 mm Hg, respectively. The systolic blood pressure showed significant increase in L-NAME, L-NAME plus SA, and L-NAME plus captopril groups compared with the control group. On the other hand, the systolic blood pressure showed no significant change in the L-NAME plus SA and captopril group compared with the control group.

### 3.2. The Impact of SA and Captopril on Vascular Biomarkers

**Figure 1:**
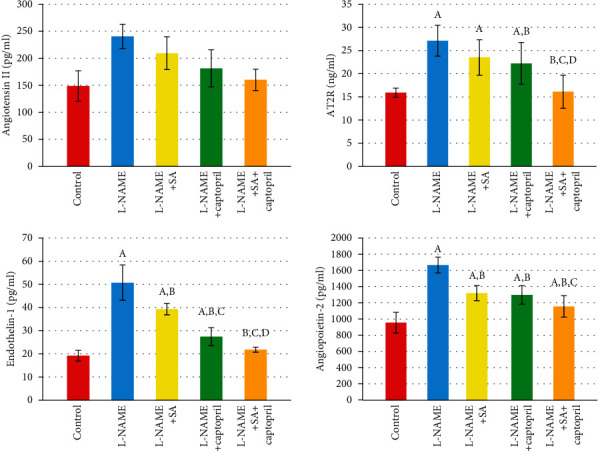
Effect of sinapic acid and captopril treatment upon angiotensin II, angiotensin II type 2 receptor (AT2R), endothelin-1, and angiopoietin-2 in different experimental groups. Values are expressed as means ± SD of 10 rats for each group. ^a^Significant difference from the control group. ^b^Significant difference from the L-NAME group. ^c^Significant difference from the L-NAME and sinapic acid (SA)group. ^d^Significant difference from the L-NAME and captopril group. *P* values ≤ 0.05 were considered significant.

Figure 1 illustrates the effect of SA and captopril on the vascular biomarker levels. By comparison to control, the group administered with L-NAME showed significantly higher levels of Ang II, AT2R, ET-1, and angiopoietin-2 in the serum. Meanwhile, by comparison to the L-NAME group, the group administered with SA exhibited significantly lower levels of ET-1 and angiopoietin-2 in the serum. By comparison to the SA group, the group administered with captopril had a significantly lower level of ET-1 in the serum (Figure 1). Furthermore, by comparison to the SA group, the group with joint SA and captopril administration showed significantly lower levels of all vascular biomarkers. Moreover, by comparison to the captopril group, the group with joint SA and captopril administration had significantly lower levels of AT2R and ET-1 in the serum. On the other hand, the SA+captopril group did not differ significantly from control in terms of the levels of Ang II, AT2R, and ET-1 in the serum (Figure 1).

### 3.3. The Impact of SA and Captopril on the Serum Lipid Profile

The alterations in serum biochemical lipid profile of the experimental groups are indicated in Table 2. By comparison to control, the L-NAME group had significantly higher levels of serum total cholesterol, triglycerides, VLDL-C, and LDL-C in the serum, but significantly lower level of HDL-C in the serum. By comparison to the L-NAME group, the group administered with SA and the group administered with SA alongside captopril showed a better lipid profile. Meanwhile, by comparison to the L-NAME group, the captopril group had significantly lower levels of total cholesterol, triglycerides, VLDL-C, and LDL-C in the serum. The sole difference between the SA group and the captopril group was a markedly lower serum LDL-C level in the former. Moreover, by comparison to the captopril group, the SA+captopril group exhibited a significantly lower level of total cholesterol. Likewise, by comparison to the SA group and the captopril group, the SA+captopril group had a significantly lower level of LDL-C, but a significantly higher HDL-C level (Table 2).

### 3.4. The Impact of SA and Captopril on Cardiac Biomarkers

**Figure 2:**
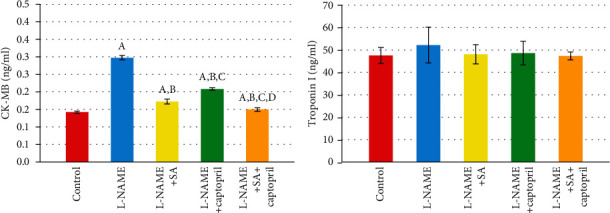
Effect of sinapic acid and captopril treatment upon CK-MB and troponin I in different experimental groups. Values are expressed as means ± SD of 10 rats for each group. ^a^Significant difference from the control group. ^b^Significant difference from the L-NAME group. ^c^Significant difference from the L-NAME and sinapic acid (SA) group. ^d^Significant difference from the L-NAME and captopril group. *P* values ≤ 0.05 were considered significant.

Figure 2 illustrates the effect of SA and captopril on the levels of cardiac biomarkers. It can be seen that, by comparison to control, the L-NAME group had a significantly higher level of CK-MB. By comparison to the L-NAME group, the groups administered with captopril, SA, and captopril combined with SA all exhibited CK-MB level modulation. Furthermore, by comparison to the SA group and the captopril group, the SA+captopril group displayed a significantly lower level of CK-MB. In fact, by comparison to control, every experimental group revealed significant alterations in the CK-MB levels. By contrast, the groups were relatively similar with regard to the levels of troponin I (Figure 2).

### 3.5. The Impact of SA and Captopril on Oxidative Stress

**Figure 3:**
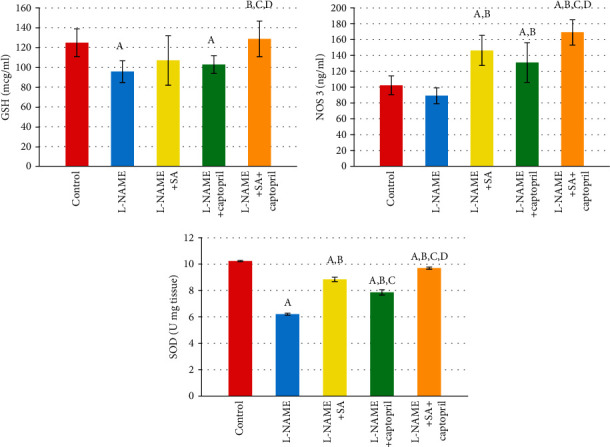
Effect of sinapic acid and captopril treatment upon oxidative stress in different experimental groups. Values are expressed as means ± SD of 10 rats for each group. ^a^Significant difference from the control group. ^b^Significant difference from the L-NAME group. ^c^Significant difference from the L-NAME and sinapic acid (SA) group. ^d^Significant difference from the L-NAME and captopril group. *P* values ≤ 0.05 were considered significant.

Figure 3 illustrates the effect of SA and captopril on the levels of oxidative biomarkers. It can be observed that, by comparison to control, the L-NAME group exhibited significantly lower levels of GSH and SOD, but not NOS3. Furthermore, by comparison to the L-NAME group, the SA group exhibited significantly higher levels of NOS3 and SOD in the serum, whereas the captopril group exhibited modulation in the level of every oxidative biomarker apart from GSH. Moreover, by comparison to the L-NAME group, the group administered with captopril alongside SA displayed modulation in the levels of every oxidative biomarker. In addition, by comparison to the captopril group, the SA group showed a significant increase in the level of SOD. By comparison with the SA group and the captopril group, the SA+captopril group had significantly higher levels of every oxidative biomarker (Figure 3).

### 3.6. Associations between Vascular Biomarkers and between Vascular Biomarkers and Cardiac and Oxidative Stress Biomarkers

In the experimental groups administered with L-NAME, L-NAME+SA, L-NAME+captopril, and L-NAME+SA+captopril, the vascular biomarkers were positively correlated with one another, as can be seen in Table 3. However, no significant correlation existed between the troponin I level and the vascular biomarkers. By contrast, the level of CK-MB was positively correlated with every vascular biomarker, and this correlation had statistical significance. Meanwhile, every oxidative biomarker was negatively correlated with every vascular biomarker and this correlation had statistical significance.

### 3.7. Associations between Cardiac Biomarkers and between Cardiac Biomarkers and Oxidative Biomarkers

In the experimental groups administered with L-NAME, L-NAME+SA, L-NAME+captopril, and L-NAME+SA+captopril, the cardiac biomarkers CK-MB and troponin I were positively correlated with one another, as can be seen in Table 4. Meanwhile, the level of CK-MB was negatively correlated with every oxidative biomarker, whereas the level of troponin I was negatively correlated solely with SOD, and both correlations were of statistical significance.

### 3.8. Regression Analysis among Research Parameters

Table 5 provides the outcomes of regression analysis. It can be observed that the levels of CK-MB were significantly positively correlated with ET-1 (*β* = 0.0024, *P* < 0.001) and significantly negatively correlated with NOS3 (*β* = −0.0004, *P* < 0.001). However, no significant correlation was established between the examined biomarkers and the levels of troponin I. Furthermore, the levels of Ang II and ET-1 were strongly predicted by CK-MB (*β* = 523.2, *P* < 0.001; *β* = 177.9, *P* < 0.001), confirming that a significant correlation existed between increased levels of CK-MB and increased levels of Ang II and ET-1 (Table 5).

### 3.9. Intergroup Linear Trend Analysis

The CK-MB level exhibited a declining linear trend of statistical significance (*P* < 0.001) across the three groups. Similarly, with regard to the lipid profiles, the levels of LDL-C, TG, total cholesterol, and VLDL-C displayed a declining linear trend of statistical significance. On the other hand, a growing linear trend of statistical significance was displayed by the level of HDL-C, as well as by the levels of GSH, NOS, and SOD. By contrast, the levels of Ang II receptors 2 and angiopoietin-II manifested a declining linear trend of statistical significance, as can be seen in Table 6.

### 3.10. Ang II Receptor Gene Expression

**Figure 4:**
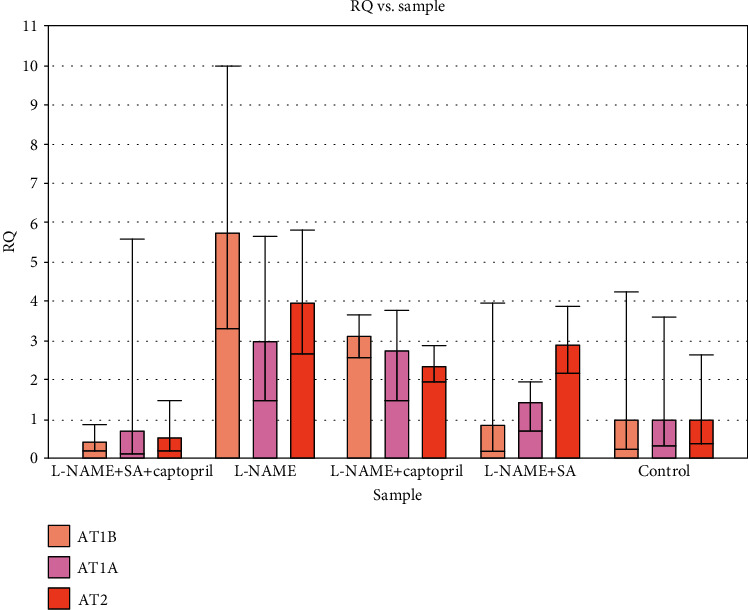
Gene expression of angiotensin II receptors (AT1A, AT1B, and AT2) of treatment groups compared to nontreated control group (relative to GAPDH (RQ)), using the 2^-*ΔΔ*CT^ method.

PCR conditions were optimised in order to validate the effectiveness and specificity of amplification of every gene expression of RT-qPCR array. The 2^-*ΔΔ*Ct^ technique was applied to contrast Ct values among treated and untreated control to enable assessment of gene expression (in relation to GAPDH) profile modulations in the L-NAME group, the L-NAME and captopril group, the L-NAME and SA group, and the L-NAME and combined SA and captopril. The relative amount of real-time qPCR was analysed and revealed that the expression of the AT1A gene increased 2.89-fold in the L-NAME group, 2.73-fold in the L-NAME and captopril group, and 1.41-fold in the L-NAME and SA group, whereas it decreased 0.74-fold in the L-NAME and combined SA and captopril group. Regarding the expression of the AT1B gene, it increased 5.74-fold in the L-NAME group and 3.06-fold in the L-NAME and captopril group, whereas it decreased 0.85-fold in the L-NAME and SA group and 0.4-fold in the L-NAME and combined SA and captopril group. Furthermore, AT2 gene expression increased 3.94-fold in the L-NAME group, 2.39-fold in the L-NAME and captopril group, and 2.9-fold in the L-NAME and SA group, while it decreased 0.54-fold in the L-NAME and combined SA and captopril group. Based on these findings, it was concluded that administration of L-NAME and combined SA and captopril minimised the expression of all genes, while the other treatments enhanced gene expression (Figure 4).

### 3.11. Heart Histopathology

**Figure 5:**
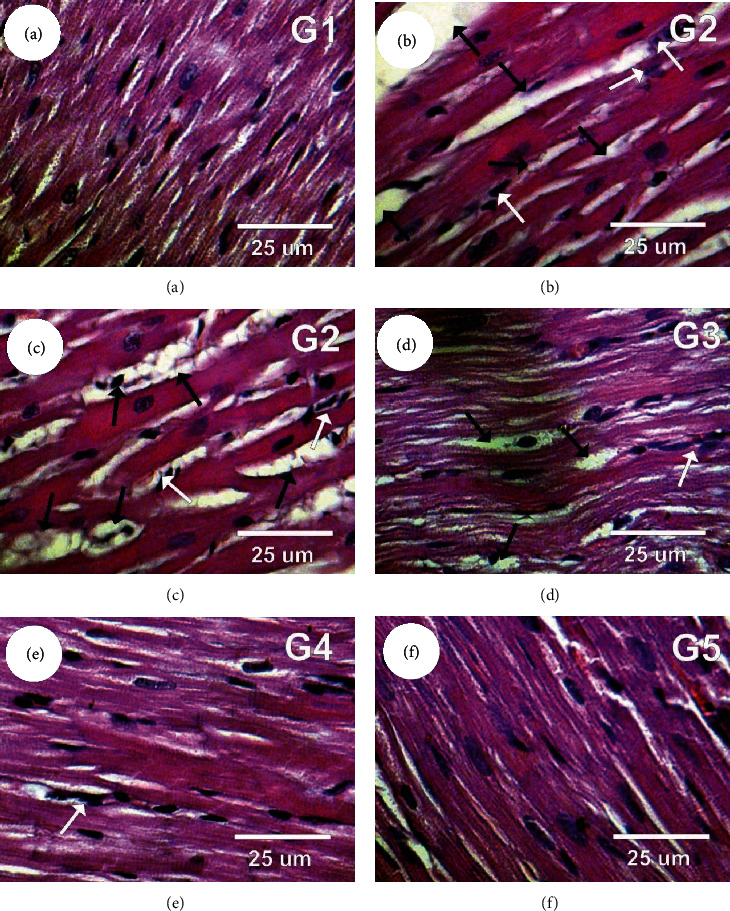
Photomicrographs of rat heart sections in the different experimental groups stained with haematoxylin and eosin. (a) Left ventricular section in the control group showing normal myofibrillar structure with striations. (b, c) Left ventricular sections in the L-NAME group (G2) revealed marked myocardial hypertrophy (black arrows) and nuclear pyknosis (white arrows). (d) Left ventricular sections in the L-NAME+SA (G3) group revealed moderate tissue injury with moderate myocardial hypertrophy (black arrows) and mild cytoplasmic vacuoles with nuclear pyknosis (white arrows). (e) Left ventricular sections in the L-NAME+captopril (G4) group revealed mild myocardial hypertrophy. (f) Heart sections in the L-NAME+SA+captopril (G5) group revealed normal myofibrillar structure with striations as in the control group.

Heart (left ventricular) sections in control (G1) group revealed a normal myofibrillar structure with striations, branched appearance, and continuity with adjacent myofibrils (Figure 5(a)). In contrast, left ventricular sections in L-NAME-induced hypertension group (G2) revealed severe hydrophobic changes of myofibrillar structure with striations, marked myocardial hypertrophy, marked cytoplasmic vacuoles, nuclear pyknosis, and focal haemorrhage (Figures 5(b) and 5(c)). On the other hand, left ventricular sections in treated hypertension with the sinapic acid (L-NAME+SA) (G3) group revealed moderate tissue injury with moderate myocardial hypertrophy, mild cytoplasmic vacuoles with nuclear pyknosis, and mild cytoplasmic vacuoles while heart sections in treated hypertension with the captopril (L-NAME+captopril) (G4) group revealed mild myocardial hypertrophy, mild myofibrillar structure with striations, branched appearance, and a few cytoplasmic vacuoles (Figures 5(d) and 5(e), respectively). In contrast; left ventricular sections in treated hypertension with the sinapic acid and captopril (L-NAME+SA+captopril) (G5) group revealed normal myofibrillar structure with striations as in control group (Figure 5(f)).

## 4. Discussion

There are more than one billion individuals suffering from hypertension, making this condition a worldwide public health concern. A chronic disease of high prevalence, hypertension is the main avoidable risk factor for myocardial infarction, heart failure, and peripheral vascular disease, being responsible for a 54% stroke-related mortality rate and a 47% mortality rate associated with coronary heart disease at a global level [2, 40, 41]. Although different types of medication are available to successfully manage hypertension and prevent CVD, they have negative side-effects that frequently restrict their usage. Hence, this work sought to assess the treatment potential of SA and captopril by investigating their effect on oxidative stress and CVD in rats with hypertension.

Damage to the myocardium can be successfully detected based on cardiac troponin I and troponin T, which are also helpful for prognostic purposes and determining the level of risk associated with different patients with acute myocardial infarction [42]. Therefore, these two troponins, and particularly troponin I, have been established as the “gold standard” for assessing cases of acute myocardial infarction. A regulatory complex comprising three protein subunits with different gene encoding, troponin is found on the thin filament of the myocardial contractile mechanism. It is the main biomarker for diagnosing myocardial infarction [43] because increase in its level is exclusively indicative of cardiac damage, whereas increase in CK-MB is not [9, 16]. Sorodoc et al. [44] reported on a case where all three aspects for diagnosing acute myocardial infarction were present, namely, clinical symptoms, electrocardiographic alterations, and increased levels of the myocardial enzymes CK and CK-MB. Furthermore, this toxic pathology has also been occasionally associated with isolated increase in the levels of enzymes.

The findings of the present work revealed that, by comparison to control, the L-NAME group exhibited a significantly higher level of CK-MB in the serum, whereas the level of troponin I in the serum did not alter markedly. On the other hand, the SA group showed a significantly lower level of CK-MB in the serum than the L-NAME group. Meanwhile, by comparison to the L-NAME group, the captopril group and the captopril+SA group exhibited modulation of the levels of both troponin I and CK-MB. This was consistent with Adamcova et al. [45], who observed that hypertension was triggered by L-NAME and cardiac enzyme levels were heightened as a result. While distinguished several possible mechanisms through which the levels of troponin could be heightened, namely, necrosis, apoptosis, turnover of normal myocyte cells, release of products from proteolytic troponin breakdown, greater cellular membrane permeability caused by integrin-mediated mechanisms associated with stretching, and membrane bleb development and release [46].

The risk of cardiac disease is assessed via a series of tests known as the lipid profile and frequently carried out jointly [16]. This profile can effectively predict the probability of occurrence of a heart attack or stroke as a result of a blocked blood vessel or arterial hardening. In general, the lipid profile looks for high levels of blood cholesterol, which are indicative of cardiac disease progression [47]; high levels of cholesterol in the blood and in tissues, which are indicative of cardiovascular damage [48]; high levels of glycerides, which can trigger pancreatic inflammation; and high levels of triglycerides, which can underpin the risk of cardiac disease.

The results of this work revealed that, by comparison to control, the L-NAME group exhibited significantly higher levels of total cholesterol, triglycerides, VLDL-C, and LDL-C in the serum, but significantly lower levels of HDL-C in the serum. However, by comparison to the L-NAME group, the groups administered with SA (i.e., the L-NAME and SA group and the L-NAME and combined SA and captopril group) displayed a better lipid profile, with diminished serum levels of total cholesterol, triglycerides, VLDL-C, and LDL-C, and improved serum levels of HDL-C.

The risk of hypertension-related CVD is elevated by increased levels of total cholesterol, which heighten predilection towards the condition called hypercholesterolemia. Extensive epidemiologic research has shown that this condition is more prevalent among hypertensive patients [47, 49]. In the present work, the levels of total cholesterol in rats with hypertension were reduced by SA administration. Roy et al. and Silambarasan et al. [50] demonstrated that sinapic acid reduced the activity of HMG-CoA reductase (the rate-controlling enzyme in the cholesterol synthesis pathway) in the liver and serum.

The increased flux of fatty acids and dysfunctional elimination of VLDL from the plasma may underpin the mechanism through which the levels of triglycerides rise as a result of hypertension. Triglycerides are transported from the liver to other tissues primarily by VLDL-C. By contrast, the liver does not produce LDL-C, which instead appears to emerge as a VLDL-C derivative after lipoprotein lipase partially eliminates triglycerides [51]. As proposed by Casazza et al. [52], a reduction in the lipoprotein lipase activity, leading to reduced triglyceride assimilation from the circulatory system, might explain the noted rise in the levels of triglycerides. The present study found that, in the L-NAME group, the levels of triglycerides were favourably influenced by SA, providing evidence that SA is capable of reducing the levels of lipids. This finding is consistent with the results of Silambarasan et al., who observed that the hepatic and renal levels of total cholesterol in l-NAME rats with spontaneous hypertension were diminished after SA administration. The study by Zych et al. [53] generated similar findings as well, with SA not only decreasing the levels of total cholesterol, triglycerides, and HOMA-IR index but also normalising certain serum markers of antioxidative capability and oxidative damage disrupted by oestrogen insufficiency in ovariectomised rats. Likewise, Gulcin [54] and Wilson et al. [55] claimed that the levels of total cholesterol, triglycerides, LDL-C, and VLDL-C in the serum of rats with diabetes were significantly lowered by SA, while the level of HDL-C was heightened by SA.

Ang II ensures ongoing contraction in the cells of vascular smooth muscle, intensifying vascular resistance, and, implicitly, raising the blood pressure. Thus, Ang II provides blood pressure modulation. The angiotensin-converting enzyme (ACE) is a major enzyme in charge of preventing fluctuations in Ang II levels. It is important for regulating the blood pressure because it undertakes the conversion of Ang I into Ang II. Ang II type one receptor (AT1R) and Ang II type two receptor (AT2R) are the two primary receptor types that are activated by Ang II. The former has broad expression and facilitates the majority of inflammatory Ang II effects, while the latter has more limited expression, stimulates vessels to dilate, and works against inflammation [56]. Furthermore, AT1R occurs in greater abundance and therefore tends to conceal the effects of AT2R. One argument is that unopposed AT2R stimulation is achieved by Angiotensin II Receptor Blockers (ARBs) by expanding Ang II availability to enable action mediation and attach to the beneficial AT2R. Essential hypertension is managed primarily with AT1R blockers [57], which are an important category of drugs targeting hypertension. Additionally, other CVDs are treated with AT1R blockers as well, particularly when they occur alongside diabetes and kidney disease.

The present work found that the L-NAME group was associated with significantly increased levels of Ang II, AT2R, ET-1, and angiopoietin-2 in the serum. By comparison to this group, the SA group exhibited significantly lower levels of ET-1 and angiopoietin-2 in the serum. Furthermore, by comparison to the SA group, the SA+captopril group displayed significantly lower levels of every vascular biomarker, while by comparison to the captopril group, the SA+captopril group had significantly lower levels of AT2R and ET-1 in the serum. Akpinar et al. [58] and Hiremath et al. [59] previously argued that perivascular inflammation followed by myocardial fibrosis was promoted by the production of ROS via Ang II.

An efficient and competitive ACE-I, captopril is capable of reducing Ang II synthesis by acting as an antagonist to enzyme-converting angiotensin in plasma, tissue, and atherosclerotic plaque, as well as diminishing water-sodium retention, disrupting bradykinin breakdown, and regulating blood pressure by suppressing the production of aldosterone and thus inducing the dilation of the coronary and peripheral arteries [60, 61]. When there is a lack of equilibrium between the ROS and antioxidant levels, the “redox” status of cells becomes imbalanced, which gives rise to oxidative stress [62, 63]. In the external environment, ROS occur in pollutants, tobacco smoke, and radiation. Additionally, different mitochondrial pathways can also lead to internal production of ROS [64, 65]. Failure of antioxidants to counteract free radicals in excessive abundance enables the latter to damage fatty tissue, DNA, and proteins. This can result in the onset of various disorders, including diabetes, atherosclerosis, Alzheimer's disease, Parkinson's disease, inflammation, hypertension, and cardiac disease.

The findings of this work revealed that, by comparison to the control group, the L-NAME group had significantly lower levels of SOD, NOS3, and GSH, which contributed to the development of hypertension. On the other hand, by comparison to the L-NAME group, the L-NAME and SA group, the L-NAME and captopril group, and the L-NAME and combined SA and captopril group had significantly higher levels of SOD, NOS3, and GSH.

As noted by Abidi et al. [66], the tripeptide GSH exhibits a robust effect against oxidants and plays a key role in the elimination of superoxide radicals, Peroxyl radicals and singlet oxygen. In the present work, the neutralisation of free radical production might have been the reason why the L-NAME group had reduced GSH levels. By contrast, the L-NAME and SA group displayed improved levels of nonenzymatic antioxidants. Hypertension is often accompanied by high blood pressure and hyperlipidemia, so much so that it has led to suggestions that changes in the lipid metabolism might be brought about by the actual high blood pressure, thus causing the development of anomalies [38].

Blood pressure increases in a volume-based manner when NO is chronically suppressed, manifesting physiologically and pathologically similar to essential hypertension. Furthermore, as observed by [27], arterial hypertension and vasoconstriction occur when NO biosynthesis is acutely suppressed as a result of the L-arginine analog L-NAME being administered in vivo. It appears that modifications in lipid metabolism unfold with the participation of L-NAME suppression of NO synthase. This has been reported to elevate the levels of cholesterol in rat serum and plasma [67] and to cause endothelium dysfunction in rabbits with hypercholesterolemia [68].

Peroxynitrite (ONOO2) is the product of the reaction between endogenous NO and superoxide and can oxidise or nitrate different biological substrates [69]. In the present work, it was found that, besides lowering the levels of H2O2-induced intracellular ROS, SA also made cells more viable and enhanced the levels of NO in H2O2-induced endothelial cells. This suggests that SA may have a safeguarding effect on endothelial cells. Cells protect themselves against oxidative damage primarily based on free radical-scavenging enzymes (e.g., SOD), which break down superoxide and hydrogen peroxide, and yield the hydroxyl radical of greater reactivity through interaction [70]. The findings obtained in this work are consistent with those of Silambarasan et al., who reported that L-NAME rats had significantly lower levels of SOD, CAT, and GPx in the heart and aorta. Meanwhile, a different study on L-NAME rats with hypertension provided evidence that SA eliminated free radicals, diminished FFA levels, and enhanced the levels of phospholipids, thus having a tissue protective effect against lipid peroxidation [29]. Moreover, the current findings are also in line with those obtained by Roy and Prince [22] in their investigation of the ability of SA to protect against cardiac hypertrophy, dyslipidaemia, and electrocardiogram changes in rats with myocardial infarction triggered by isoproterenol. The efficiency of SA against oxidants might stem from the capacity of derivatives of hydroxycinnamic acid to counteract free radicals by freeing up a hydrogen atom and forming a phenoxy radical. Stabilisation of the latter is achieved by a conjugated system comprising an arene and an alkenyl carboxylate side chain [71, 72].

Left ventricular hypertrophy, abnormal systolic and diastolic function, and even overt cardiac failure are among the heart structure or function irregularities that are often presented by hypertensive individuals. Additionally, these individuals may suffer from coronary heart disease and are more likely to develop arrhythmia and to die unexpectedly. An indicator of and contributor to coronary events, stroke, heart failure, peripheral arterial disease, and death due to cardiovascular conditions in hypertensive individuals is the atypical mass expansion in the left ventricle known as left ventricular hypertrophy (LVH) [9, 16, 22]. The L-NAME group in the present work exhibited radical hydrophobic alterations in the myofibrillar structure with striations, major myocardial hypertrophy, extensive vacuoles in the cytoplasm, nuclear pyknosis, and focal haemorrhage in the left ventricle. As noted by several authors, an association exists between cardiac hypertrophy and hypertension, augmented fibrosis, collagen accumulation, and diminished cardiac function [9]. The L-NAME and SA experimental group displayed moderate tissue damage with moderate myocardial hypertrophy and mild cytoplasmic vacuoles alongside nuclear pyknosis. This presentation might be attributed to the ability of SA to counteract hypertension, thus alleviating hypertrophy triggered by pressure load. By contrast, the L-NAME and captopril experimental group presented mild myocardial hypertrophy, mild myofibrillar structure with striations, ramified appearance, and a limited number of vacuoles in the cytoplasm. Numerous hypertensive individuals have myocardial fibrosis, which is believed to be the last common pathway that eventually leads to permanent cardiac failure [73]. The present work concluded that joint administration of SA and captopril to rats with hypertension ameliorated the high blood pressure, enhanced cardiovascular function, hindered the onset of hyperlipidemia, and decreased oxidative stress and myocardial hypertrophy. It could thus be implied that, when administered alongside captopril, SA could have a beneficial effect for the management of cardiac disease with hypertension.

## Figures and Tables

**Table 1:** The genes subjected to amplification and the primer sequences.

Manufacturer	Gene	Primers
BioNira (USA)	AT1A	F-CTCAAGCCTGTCTACGAAAATGAG R-TAGATCCTGAGGCAGGGTGAAT

BioNira (USA)	AT1B	F-CTTTCCTACCGCCCTTCAGATA R-TGAGTGCTTTCTCTGCTTCAAC

BioNira (USA)	AT2	F-ACCTTTTGAACATGGTGCTTTG R-GTTCTCTGGGTCTGTTTGCTC

**Table 2:** Serum lipid profile levels in different studied groups.

Parameters	Groups				
	Control group	L-NAME group	L-NAME+SA group	L-NAME+captopril group	L-NAME+SA+captopril group
Total cholesterol (mg/dl)	78.6 ± 5.63	151.2 ± 9.15^a^	86.8 ± 4.82^b^	96.5 ± 10.11^a,b^	80.7 ± 5.66^b,d^
Triglycerides (mg/dl)	62.8 ± 7.12	179.3 ± 9.6^a^	68.1 ± 6.89^b^	73.8 ± 7.66^a,b^	64.3 ± 8.22^b^
HDL-C (mg/dl)	50.6 ± 4	27.1 ± 3.59^a^	32.7 ± 5.12^a,b^	31.5 ± 4.15^a^	40.8 ± 3.25^a,b,c,d^
VLDL-C (mg/dl)	12.6 ± 1.42	35.9 ± 1.92^a^	13.6 ± 1.38^b^	14.8 ± 1.53^a,b^	12.9 ± 1.64^b^
LDL-C (mg/dl)	17.7 ± 3.02	88.2 ± 9.67^a^	40.4 ± 4.32^a,b^	50.2 ± 7.51^a,b,c^	28.1 ± 4.84^a,b,c,d^

Values are represented as mean ± SD of 10 rats for each group. ^a^Significant difference from the control group. ^b^Significant difference from the L-NAME group. ^c^Significant difference from the L-NAME and sinapic acid (SA) group. ^d^Significant difference from the L-NAME and captopril group. *P* values ≤ 0.05 were considered significant.

**Table 3:** Correlations of vascular biomarkers with cardiac, oxidative stress biomarkers, and each other.

Vascular biomarkers	Ang II (pg/ml)	AT2R (ng/ml)	ET-1 (pg/ml)	Angiopoietin-2 (pg/ml)
AT2R (ng/ml)	0.535∗∗	—		
ET-1 (pg/ml)	0.680∗∗	0.656∗∗	—	
Angiopoietin-2 (pg/ml)	0.641∗∗	0.504∗∗	0.771∗∗	—
CK-MB (ng/ml)	0.616∗∗	0.627∗∗	0.746∗∗	0.831∗∗
Troponin I (ng/ml)	0.227	0.225	0.223	0.287
GSH (mcg/ml)	-0.415∗∗	-0.454∗∗	-0.444∗∗	-0.379∗
NOS3 (ng/ml)	-0.571∗∗	-0.509∗∗	-0.666∗∗	-0.708∗∗
SOD (U mg tissue)	-0.630∗∗	-0.625∗∗	-0.732∗∗	-0.843∗∗

Values are expressed as correlation coefficients (*r*). Ang II: angiotensin II; AT2R: angiotensin II type 2 receptor; ET-1: endothelin-1; CK-MB: creatine kinase-MB; GSH: reduced glutathione; NOS3: nitric oxide synthase 3; SOD: superoxide dismutase. ∗Correlation was significant at *P* ≤ 0.05. ∗∗Correlation was significant at *P* ≤ 0.01.

**Table 4:** Correlations of cardiac biomarkers with oxidative biomarkers.

Vascular biomarkers	CK-MB (ng/ml)	Troponin I (ng/ml)
Troponin I (ng/ml)	0.332∗	—
GSH (mcg/ml)	-0.499∗∗	-0.058
NO synthase 3 (ng/ml)	-0.844∗∗	-0.181
SOD (U mg tissue)	-0.978∗∗	-0.33∗

Values are expressed as correlation coefficients (*r*). GSH: reduced glutathione; NOS3: nitric oxide synthase; SOD: superoxide dismutase; CK-MB: creatine kinase-MB. ∗Correlation was significant at *P* ≤ 0.05. ∗∗Correlation was significant at *P* ≤ 0.01.

**Table 5:** Regression analysis between different investigated parameters.

	CK-MB (ng/ml)	Troponin I (ng/ml)	Ang II (pg/ml)	Endothelin-1 (pg/ml)
Predictors	Estimates	Estimates	Estimates	Estimates
Ang II (pg/ml)	0.0002	0.0113	—	—
Endothelin-1 (pg/mL)	0.0024∗∗∗	0.0796	—	—
GSH (mcg/ml)	-0.0004	0.0156	-0.1556	-0.0591
NOS3 (ng/ml)	-0.0004∗∗	-0.0033	0.1619	0.0406
CK-MB (ng/ml)	—	—	523.1760∗∗∗	177.8672∗∗∗
Troponin I (ng/ml)	—	—	-0.0976	-0.0744
*R* ^2^/*R*^2^ adjusted	0.739/0.716	0.063/-0.020	0.468/0.421	0.659/0.628

Values are expressed as standardized coefficients (*β*). GSH: reduced glutathione; NOS3: nitric oxide synthase 3; CK-MB: creatine kinase-MB; Ang II: angiotensin II. ∗∗*P* < 0.01. ∗∗∗*P* < 0.001.

**Table 6:** Linear trend analysis across different investigated groups.

	L-NAME+captopril	L-NAME+SA	L-NAME+SA+captopril	*P*
Cardiac markers				
CK-MB (ng/ml)	0.21 ± 0.00	0.17 ± 0.01	0.15 ± 0.01	<0.001
Troponin I (ng/ml)	48.6 ± 5.27	48.1 ± 4.31	47.3 ± 1.74	0.496
Lipid profile				
HDL-C (mg/dl)	31.5 ± 4.15	32.7 ± 5.12	40.8 ± 3.25	<0.001
LDL-C (mg dl)	50.2 ± 7.51	40.4 ± 4.32	28.1 ± 4.84	<0.001
TG (mg/dl)	73.8 ± 7.66	68.1 ± 6.89	64.3 ± 8.22	0.008
Total cholesterol (mg/dl)	96.5 ± 10.1	86.8 ± 4.82	80.7 ± 5.66	<0.001
VLDL-C (mg dl)	14.8 ± 1.53	13.6 ± 1.38	12.9 ± 1.64	0.008
Oxidative markers				
GSH (mcg/ml)	103 ± 9.19	107 ± 25.1	129 ± 17.8	0.004
NO synthase 3 (ng/ml)	131 ± 25.0	146 ± 18.9	169 ± 15.9	<0.001
SOD (U mg tissue)	7.86 ± 0.21	8.85 ± 0.16	9.70 ± 0.08	<0.001
Vascular markers				
Angiotensin II (pg/ml)	181 ± 34.5	209 ± 30.5	160 ± 19.8	0.181
Angiotensin II receptor 2 (ng/ml)	22.2 ± 4.52	23.5 ± 3.83	16.1 ± 3.57	0.005
Angiopoietin-II (pg/ml)	1297 ± 113	1320 ± 93.1	1156 ± 134	0.014
Endothelin-1 (pg/ml)	27.4 ± 3.85	39.3 ± 2.45	21.8 ± 1.09	0.117

Values are expressed as means ± SD. *P*: *P* value for the linear trend: L-NAME+captropril < L-NAME+SA < L-NAME+SA+captropril. *P* values ≤ 0.05 were considered significant.

## Data Availability

The data used to support the findings of this study are available from the corresponding author upon request.
